# Dysregulation of the Environmental Sensor Aryl Hydrocarbon Receptor Affects Differentiation of Human Colon Organoids

**DOI:** 10.1016/j.jcmgh.2023.11.002

**Published:** 2023-11-07

**Authors:** Anke Liebert, Michael Shapiro, Muralidhara Rao Maradana, Ying Li, Nick Powell, Matthias Zilbauer, Brigitta Stockinger

**Affiliations:** The Francis Crick Institute, AHR Immunity Laboratory, London, United Kingdom; Faculty of Medicine, Department of Metabolism, Digestion and Reproduction, Imperial College, London, United Kingdom; School of Clinical Medicine, Department of Paediatrics, University of Cambridge, Cambridge, United Kingdom; The Francis Crick Institute, AHR Immunity Laboratory, London, United Kingdom

Gut disorders such, as inflammatory bowel disease (IBD), have increased dramatically in the past 50 years, driven by incompletely defined environmental factors including diet, with reduced intake of substances such as indole compounds, which function as aryl hydrocarbon receptor (AHR) ligands. The AHR resides in the cytoplasm complexed with chaperones, and upon ligand binding translocates to the nucleus where it dimerizes with its partner aryl hydrocarbon nuclear translocator, and initiates the transcription of a wide range of target genes. In the intestinal milieu, AHR ligands are derived from the metabolism of dietary compounds as well as from tryptophan metabolism by the microbiota (reviewed by Stockinger et al^1^).

Studies in mouse models have established important functions for AHR in intestinal hematopoietic as well as nonhematopoietic cell types.^1^ AHR safeguards a regulated regenerative response upon injury, thus placing it in a crucial position to maintain the balance between regeneration and malignant transformation.^2^

Considering its evolutionary conservation, it is likely that AHR plays similar roles in human beings, supported by its identification as a susceptibility locus in IBD, particularly in ulcerative colitis (UC).^3^

We investigated the role of AHR in intestinal organoids from either healthy human donors or UC patients (Supplementary Table 1) and conducted RNA sequencing (RNAseq) comparisons of organoids with wild-type AHR or AHR rendered nonfunctional by administration of a potent antagonist or CRISPR-Cas9 (CRISPR associated protein 9) mediated knockout.

The AHR pathway in organoids was activated by the high-affinity AHR ligand 6-formylindolo(3,2-b)carbazole (FICZ), leading to the expression of the canonical AHR target genes of cytochrome P450 family 1, subfamily A, polypeptide 1 and B1 (*CYP1A1*, *CYP1B1*) and aryl hydrocarbon receptor repressor (*AHRR*) (Figure 1*A*), which reached maximum at 4 hours of treatment (Figure 1*B*), whereas enzyme activity for CYP1A1/B1 peaked at 6 hours (Figure 1*C*).

**Figure 1:**
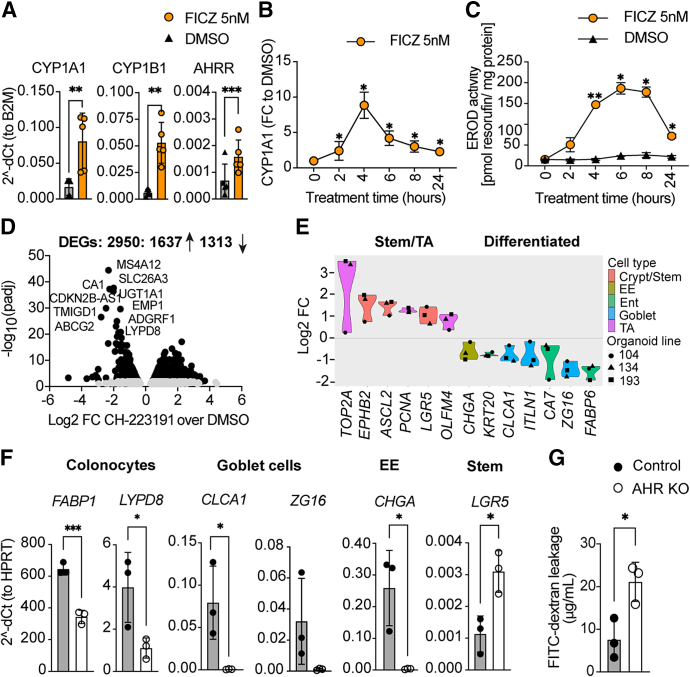
**AHR inhibition impairs organoid differentiation.** (*A*) Quantitative polymerase chain reaction (qPCR) expression of AHR target genes in 6-formylindolo(3,2-b)carbazole (FICZ)-treated organoids. (*B*) CYP1A1 expression after FICZ treatment, normalized to dimethyl sulfoxide (DMSO) control. (*C*) Ethoxyresorufin-O-deethylase (EROD) assay after FICZ treatment. (*D*) Volcano plot of DEGs. (*E*) Marker gene expression for epithelial cell subtypes. (*F*) qPCR expression of epithelial marker genes of control and AHR KO organoids. (*G*) Fluorescein isothiocyanate (FITC)-dextran transepithelial permeability on air-liquid-interface (ALI) monolayer cultures. All experiments were performed at least twice with 3–5 samples per genotype. ∗*P* < .05, ∗∗*P* < .01, and ∗∗∗*P* < .001. EE, enteroendocrine cells; Ent, enterocytes; TA, transit amplifying cells; Stem, stem cells. AHRR, aryl hydrocarbon receptor repressor; B2M, Beta-2-microglobulin; dCt, delta Ct; FC, fold change; HPRT, Hypoxanthine phosphoriboslyltransferase 1.

AHR is required for maturation of the intestinal epithelium from intestinal stem cells in mice.^2^ To investigate this in human epithelium, we performed RNAseq comparing untreated organoids with those treated with the AHR inhibitor CH-223191 to mimic an AHR-deficient state (Supplementary Figure 1*A*). Organoids were induced to differentiate, with marker gene expression indicating the state of differentiation (Supplementary Figure 1*B*). The kinetics of inhibition by CH-223191 as well as the titration of the inhibitor are shown in Supplementary Figure 1*C* and *D*.

Principal component analysis of the RNAseq data generated from organoids kept under stem cell (WENR) or differentiating conditions for 4 days (4d ENR) revealed differences between the transcriptomes of control and AHR inhibitor–treated organoids, indicative of incomplete maturation without AHR activity (Supplementary Figure 1*E*). DESeq analysis revealed 2950 differentially expressed genes (DEGs) between AHR-sufficient and AHR-deficient organoids under differentiating conditions (Figure 1*D*). The DEGs affected by AHR-inhibitor treatment correlated positively with the signature of transit-amplifying, stem cells and tuft cells from transcriptomic data of healthy human colon,^4^ but were enriched negatively for subsets of mature epithelial cells (Figure 1*E*, Supplementary Figure 1*F*). Inhibition of AHR in organoids kept under stem cell conditions had less effect on gene expression with only 5 DEGs (Supplementary Methods section).

To exclude possible side effects of the AHR inhibitor or an incomplete inhibition, we generated several independent clones of AHR knockout (KO) organoids via CRISPR-Cas9 mediated gene editing^5^ (Supplementary Figure 2*A*), which resulted in a truncated protein as verified by western blot (Supplementary Figure 2*B*). The edited clones did not up-regulate AHR target genes after treatment with ligand (Supplementary Figure 2*C*) and showed impaired differentiation with low expression of marker genes for mature colonic epithelial cells, goblet cells, and enteroendocrine cells, as well as considerably higher expression of the stem cell marker gene *LGR5* (Figure 1*F*). AHR contributes to tight junction integrity,^6^ and AHR KO organoid monolayers had a higher fluorescein isothiocyanate (FITC)–dextran flux, indicating a barrier defect (Figure 1*G*).

IPA Ingenuity software (QIAGEN) predicted members of the MYC/MAX/MAD superfamily of transcription factors as most significantly activated upstream in the 4d ENR AHR inhibitor–treated organoids, whereas transcription factors controlling cell-cycle progression, such as Nuclear protein 1 (NUPR1) and Tumor protein 53 (TP53), or epithelial differentiation, such as HNF1 homeobox A (HNF1A) and Hepatocyte nuclear factor 4 alpha (HNF4A), were predicted to be inhibited in the AHR-deficient organoids (Figure 2*A*).

**Figure 2:**
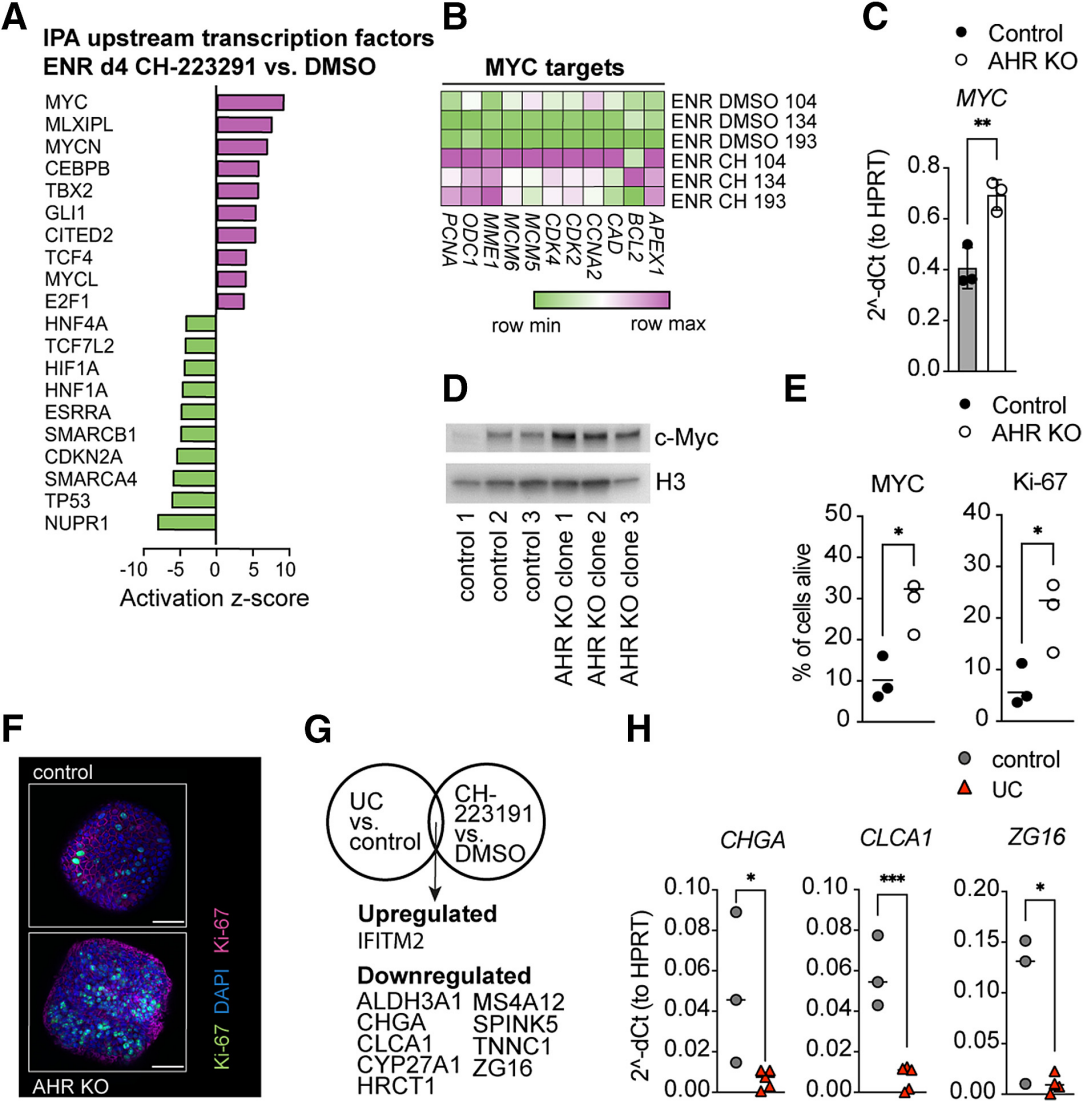
**Increased MYC activity and overlap with UC in AHR deficiency.** (*A*) Activation z-score transcription factors upstream of DEGs from the RNAseq data set. (*B*) Heatmap of MYC target genes. (*C*) Quantitative polymerase chain reaction (qPCR) expression of MYC in control and AHR KO organoids. (*D*) MYC western blot of control and AHR KO organoids. (*E*) Percentage of MYC and KI-67–positive cells. (*F*) Representative images showing KI-67 (green) expression in control and AHR KO organoids. *Scale bar*: 50 μmol/L. (*G*) Venn diagram showing overlapping genes commonly up-regulated or down-regulated (≥1.5 fold) in UC patient organoids vs controls and AHR-deficient organoids. (*H*) qPCR expression of mature epithelial marker genes of control and UC patient organoids. DAPI, 4′,6-diamidino-2-phenylindole; dCt, delta Ct; DMSO, dimethyl sulfoxide; ENR, differentiation medium; HPRT, Hypoxanthine phosphoribosyltransferase 1.

Higher expression of MYC signature genes was also reflected in our RNAseq data set (Figure 2*B*) and verified by quantitative polymerase chain reaction (Figure 2*C*) and western blot (Figure 2*D*). MYC is among the signature of proliferative genes commonly up-regulated in the inflamed epithelium of IBD patients.^3^

Furthermore, AHR-deficient organoids had a higher percentage of MYC and Ki-67–positive cells (Figure 2*E* and *F*), suggesting an increase of actively cycling cells in the AHR KO organoids. MYC might be a direct target of AHR in human colon organoids, as supported by evidence from chromosome immunoprecipitation data in human cell lines.^7^

Recent studies have identified transcriptional signatures specific for IBD^4^^,^^8^^,^^9^ and showed that differences between patients with UC and non-IBD controls are partially maintained in organoid culture.^10^ Overlaying our RNAseq data set with data sets from ulcerative colitis vs. control patients, gene set enrichment analysis identified a significant overlap of DEGs between control and AHR inhibitor–treated organoids with genes down-regulated in UC patient-derived organoids (Supplementary Figure 3*A*) and genes of the epithelium of UC patients from both inflamed and noninflamed tissue (Supplementary Figure 3*B*). Specific genes commonly up-regulated or down-regulated in the transcriptional profiles of differentiated UC-derived and AHR-deficient organoids were identified (Figure 2*G*). Of 11 genes analyzed by quantitative polymerase chain reaction, *CLCA1*, *CHGA*, and *ZG16* were confirmed to be down-regulated in both differentiated AHR KO and UC patient-derived organoids (Figure 2*H*, Supplementary Figure 3*C*).

All together, our data indicate that evolutionary conserved functions of AHR in intestinal epithelial cells shape the regeneration and differentiation of intestinal stem cells not only in mice, but also in humans.

## Figures and Tables

**Supplementary Figure 1:**
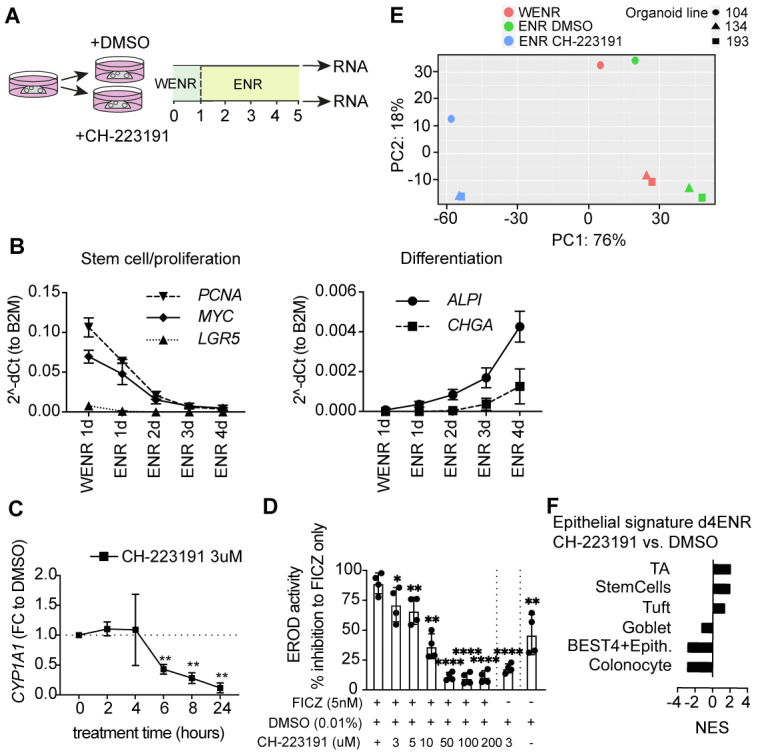
(*A*) Schematic for RNAseq of organoids treated with 3 μmol/L CH-2231910 or 0.01% dimethyl sulfoxide (DMSO) control in WENR (stem cell condition) or 4 days in ENR (differentiating condition). (*B*) Quantitative polymerase chain reaction marker gene expression for stem cell/proliferative cells (*LGR5, PCNA, MYC*) enterocytes (*ALP*I), and enteroendocrine cells (*CHGA*) under WENR and ENR conditions. (*C*) *CYP1A1* expression after CH-223191 treatment, normalized to DMSO control. (*D*) AHR-inhibitor titration for CYP1A1/B1 ethoxyresorufin-O-deethylase (EROD) activity normalized to 6-formylindolo(3,2-b)carbazole (FICZ) = 100%. (*E*) Principal component analysis of d4 ENR differentiated organoids vs organoids in WENR medium. (*F*) Enrichment plot of d4 ENR CH-223191–treated organoids compared with transcriptional signatures from colon epithelial cell subtypes. BEST4, BEST4 espressing epithelial cells; B2M, Beta-2-microglobulin; dCt, delta Ct; Epith., Epithelial cells; FC, fold change; NES, normalized enrichment score; PC, principal component; TA, transit amplifying cells.

**Supplementary Figure 2:**
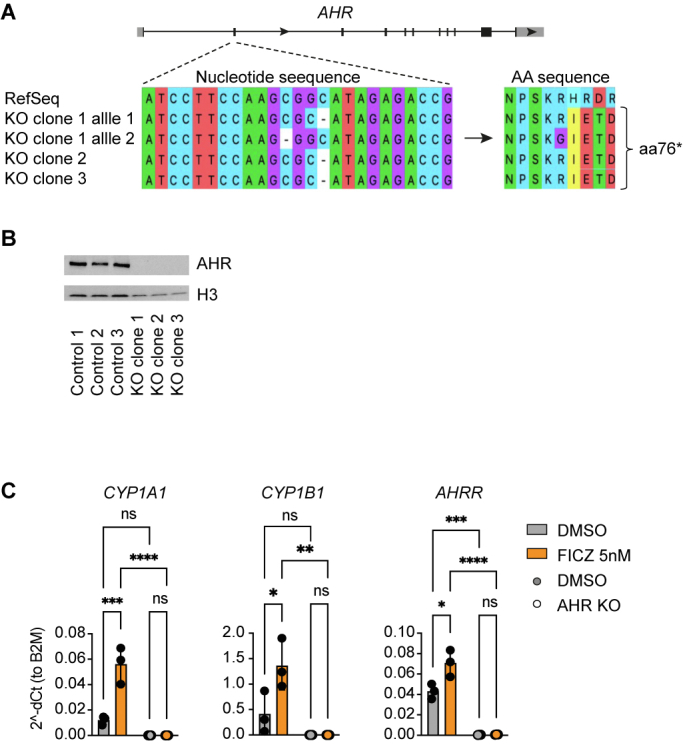
(*A*) DNA and translated amino acid (aa) sequence of AHR KO organoid clones. (*B*) Western blot image of nuclear extracts of 3 different control and AHR KO organoid clones. (*C*) Quantitative polymerase chain reaction expression data of AHR target genes in AHR KO and control colon organoids treated with 6-formylindolo(3,2-b)carbazole (FICZ) (4 h). ∗*P* < .05, ∗∗*P* < .01, and ∗∗∗*P*, aryl hydrocarbon receptor repressor; B2M, Beta-2-microglobulin; dCt, delta Ct; DMSO, dimethyl sulfoxide; H3, Histone H3.

**Supplementary Figure 3:**
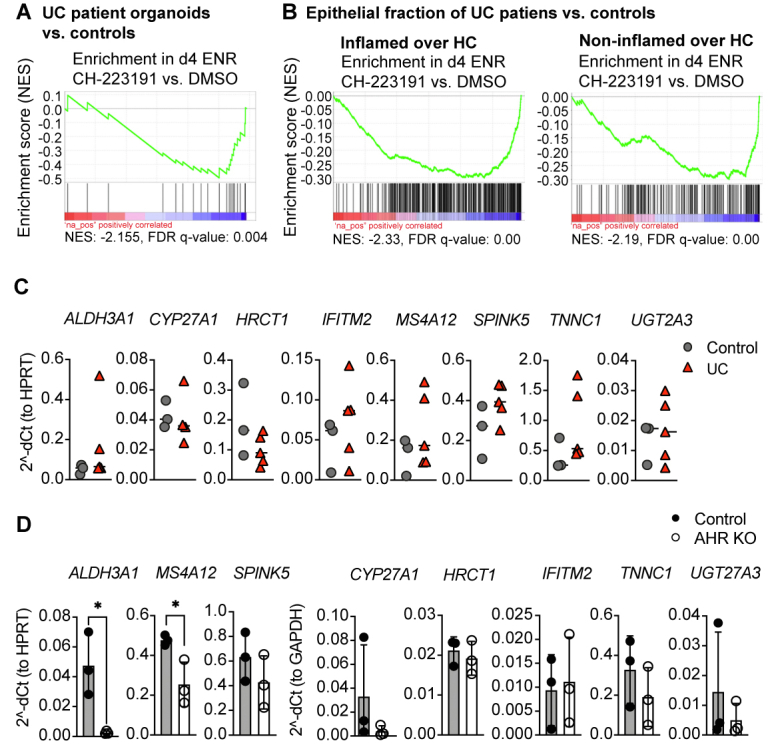
(*A*) Gene set enrichment plots of DEGs of the 4 day ENR (4d ENR) CH-223191 vs. DMSO RNAseq relative to DEGs in UC patient organoids vs. controls.^9^ (*B*) Gene set enrichment plots of DEGs of the 4d ENR CH-223191 vs. DMSO RNAseq relative to DEGs of either inflamed or noninflamed epithelium of UC patients vs healthy control (HC) samples.^8^ (*C* and *D*) Quantitative polymerase chain reaction (qPCR) expression data of overlapping genes from Figure 2*G*. (*C*) d4 ENR UC and non-IBD adult patient organoids. (*D*) 4d ENR control and AHR KO organoids. dCt, delta Ct; DMSO, dimethyl sulfoxide; FDR, false discovery rate; HPRT, Hypoxanthine phosphoribosyltransferase 1; NES, normalized enrichment score.
