# Supplementary material for: Dysregulation of the Environmental Sensor Aryl Hydrocarbon Receptor Affects Differentiation of Human Colon Organoids
Source: Cell Mol Gastroenterol Hepatol. 2023 Nov 7;17(3):507–10. doi: 10.1016/j.jcmgh.2023.11.002 (PMC10884557; doi:10.1016/j.jcmgh.2023.11.002)
Supplement: Supplementary Methods [file mmc1.docx]

**Methods**

**Human samples for intestinal organoids**

Intestinal biopsies for healthy control paediatric organoids were obtained (ethical approval REC 17/EE/0265) from children up to the age of 16 undergoing diagnostic endoscopy at Addenbrooke’s Hospital, Cambridge.

Adult human UC and control samples were obtained from the Imperial College Healthcare Tissue Bank (ICHTB). ICHTB is approved by Wales REC3 to release human material for research (22/WA/0214), and the samples for this project R20025 were issued from sub-collection reference number MET_RS_20_005 & SMT_PT_20_001.

UC disease status was confirmed by histology and control samples had macroscopically and histologically normal mucosa. Sample information is listed in Supplementary Table 1.

**Organoid culture**

Organoids were generated from colon mucosa biopsies as described^1, 2^. WENR culture medium contained advanced DMEM/F-12, 10mM HEPES, 2mM GlutaMAX, B-27, 1.25mM N-Acetylcystenine, 50ng/ml murine EGF, 100ng/ml human Noggin (Peprotech), 500nM ALK5 inhibitor A83-01, 10uM SB202190 and 10mM Nicotinamide. The medium further contained Wnt-3a- conditioned (50% v/v) and R-spondin conditioned medium (20% v/v), obtaind from the Cell Services platform at the Francis Crick Institute. For differentiating conditions ENR medium lacking Wnt-3a, SB202190 and Nicotinamide was used for 4 days and renewed daily.

**RNA extraction and q-PCR**

Total RNA from organoids was isolated from organoids using TRIzol™ Reagent (Thermo Fisher Scientific) and converted into cDNA using the High-Capacity cDNA Reverse Transcription Kit (Thermo Fisher Scientific) according to manufacturer’s instructions. Genes of interest and house-keeping genes were amplified by real-time qPCR using the TaqMan Universal PCR Master Mix on a QuantStudio 7 and the following TaqMan gene expression probes (Thermo Fisher Scientific):

AHRR (Hs01005075_m1), ALDH3A1 (Hs00167476_m1), ALPI (Hs00357579_g1), B2M (Hs99999907_m1), CHGA (Hs00900369_m1), CLCA1 (Hs00976292_m1), CYP1A1 (Hs00153120_m1), CYP1B1 (Hs00164383_m1), CYP27A1 (Hs01026016_m1), GAPDH (Hs02758991_g1), HPRT1 (Hs02800695_m1), HRCT1 (Hs02742472_s1), IFITM2 (Hs00829485_sH), LGR5 (Hs00173664_m1), LYPD8 (Hs01002757_m1), MS4A12 (Hs01113423_m1), MYC (Hs00153408_m1), PCNA (Hs00427214_g1), UGT2A3 (Hs00226904_m1), SPINK5 (Hs00928570_m1), TNNC1 (Hs00896999_g1), ZG16 (Hs00380609_m1).

**Ethoxyresorufin-O-deethylase (EROD) assay**

Organoids were incubated with 5nM FICZ, DMSO or CH-223191^3^ for different time periods and then washed with warm PBS, followed by an incubation with 2mM 7-ethoxyresorufin in sodium phosphate buffer (50 mM, pH 8.0) for 30 min at 37 °C. The reaction was stopped by fluorescamine dissolved in acetonitrile. Formation of resorufin and fluorescamine was quantified using a TECAN Spark plate reader (excitation/emission of 535/590 and 390/485 nm respectively).

**RNA sequencing**

Organoids derived from paediatric control samples were recovered from Matrigel using Cell recovery solution (Corning) and RNA was extracted using the RNeasy Plus Mini Kit (Qiagen) and converted into libraries using the KAPA mRNA HyerPrep kit (Roche) following the manufacturer’s instructions. Sequencing was performed on an Illumina HiSeq4000 platform with single-ended reads of ≥75 bp.

**Bioinformatic analysis**

Computations were performed in R, 4.2.2 (2022-10-31). Sequencing datasets are available on GEO, accession number GSE22911. Selected code, data and details on analysis are available at https://github.com/michaeldshapiro/AnkesFlyingCircusOfOrganoids.

**Generation of AHR KO organoids**

AHR KO organoids were created from a paediatric control organoid line following the protocol as described^10^. Transfection was performed using a NEPA21 electroporator (Sonidel) with 10ug pSpCas9(BB)-2A-GFP (Addgene) containing guide RNAs (CGGTCTCTATGCCGCTTGGA and AAGTCGGTCTCTATGCCGCT) targeting exon 2 of AHR or pSpCas9(BB)-2A-GFP without guides as control. Single cell organoid suspensions were sorted for live GFP expressing cells on an ARIAIII cell sorter. After 2-3 weeks single organoids were picked and seeded in Matrigel (Growth factor Reduced Basement, Corning). Putative AHR KO organoid clones were identified by qPCR expression of CYP1A1 after incubation with 5nM FICZ for 4 hours. Sequences were verified by amplicon sequencing on an llumina MiSeq platform. The Phusion™ High-Fidelity DNA Polymerase in combination with MySeq primer (Fwd: 5’-TCGTCGGCAGCGTCAGATGTGTATAAGAGACAGGCAGCGCCAACATCACCTAC-3’, Rev: 5’-GTCTCGTGGGCTCGGAGATGTGTATAAGAGACAGGACGCTGAGCCTAAGAACTGAA-3’) was used to amplify the genetic region of interest.

**ALI culture and permeability assay**

2D Air-liquid interface (ALI) colon organoid cultures were set up as previously described^12^.

Permeability of the epithelial layer of day 21 ALI cultures was tested by removing medium from the transwells and adding 4kDa FITC-dextran (Sigma) to the apical chamber in a final concentration of 1mg/ml in MEM w/out phenol red (Thermo Fisher Scientific). Transwell plates were incubated at 37 °C for 40 min and media was removed from the bottom compartment for fluorescence measurement using a TECAN Spark plate reader (excitation/emission of 490/520).

**Flow Cytometry**

Single-cell suspensions were stained with a fixable Near IR live/dead stain viability dye (Thermo Fisher Scientific) for 30 min on ice. Cells were fixed and permeabilised using the eBioscience™ Foxp3/Transcription Factor Staining Buffer Set (Thermo Fisher Scientific) following the manufacturer’s instructions. For intracellular staining, cells were incubated with FITC-anti-c-Myc (1:200, Abcam, ab223913) and APC-anti KI-67 (1:200, Invitrogen, 17-5698-82) for 1h at RT. Samples were acquired on a BD Fortessa Cytometer (BD Biosciences) and analysed using FlowJo software (v10, FlowJo LLC).

**Western blotting**

Organoids were recovered from Matrigel using Cell recovery solution (Corning) and proteins were extracted using the NE-PER™ Nuclear and Cytoplasmic Extraction Reagents (Thermo Fisher Scientific). 10-40 mg of protein was loaded and separated on 4–15% Mini-PROTEAN® TGX™ Precast gels and transferred to PVDF membranes using the Trans-Blot® Turbo™ Transfer System (all Bio-rad). After blocking the membranes were incubated with anti-AHR (1:1000, Cell Signalling, 83200), anti c-Myc (1:1000, Abcam, ab32072) or anti-Histone H3 control antibody (1:25000, Millipore) and ECL Rabbit -HRP-linked whole Ab (1:10000, GE Healthcare, NA934) as secondary antibody.

**Histology and immunostaining**

Organoids grown in Matrigel or on transwells were fixed in 4% Paraformaldehyde for 20 min at room temperature and then permeabilised with 1% Triton X-100 in PBS for 60 min and blocked with 5% donkey serum in PBS before incubation with antibody anti KI-67 (1:200, abcam, ab16667) for 12 h at 4° in 1% BSA, 0.2% Triton X-100, 0.05% Tween. Secondary staining was done for 1h at RT with Donkey anti-rabbit AF488 (1:500, Thermo Fisher Scientific, A21206), Rhodamine phallodin (1:1000, Thermo Fisher Scientific) and DAPI.

**Disclaimer**

Tissue samples were provided by the Imperial College Healthcare NHS Trust Tissue Bank funded by the National Institute for Health Research (NIHR) Biomedical Research Centre based at Imperial College Healthcare NHS Trust and Imperial College London. The views expressed are those of the author(s) and not necessarily those of the NHS, the NIHR or the Department of Health.

**References**

1. Sato T et al, Gastroenterology 2011;141:1762-72.

2. Howell KJ et al, Gastroenterology 2018;154:585-598.

3. Kim SH et al, Mol Pharmacol 2006;69:1871-8.

4. Lawrence M et al, PLoS Comput Biol 2013;9:e1003118.

5. Love MI et al, Genome Biol 2014;15:550.

6. Liberzon A et al, Cell Syst 2015;1:417-425.

7. Hao Y et al, Cell 2021;184:3573-3587 e29.

8. Parikh K et al, Nature 2019;567:49-55.

9. Dotti I et al, Gut 2017;66:2069-2079.

10. Fujii M et al, Nat Protoc 2015;10:1474-85.

11. Han H et al, EMBO J 2020;39:e104319.

12. Wang Y et al, Neural Regen Res 2015;10:2011-7.

**Legends for Supplementary figures**

**Suppl. Figure 1**

A) Schematic for RNAseq of organoids treated with 3μM CH-2231910 or 0.01% DMSO control in WENR (stem cell condition) or d4 ENR (differentiating condition). B) qPCR marker gene expression for stem cell/proliferative cells (LGR5, PCNA, MYC) enterocytes (ALPI) and enteroendocrine cells (CHGA) under WENR and ENR conditions. C) CYP1A1 expression after CH-223191 treatment, normalized to DMSO control. D) AHR inhibitor titration for CYP1A1/B1 EROD activity normalised to FICZ =100%. E) Principal component analysis of d4 ENR differentiated organoids vs. organoids in WENR medium. F) Enrichment plot of d4 ENR CH-223191 treated organoids compared to transcriptional signatures from colon epithelial cell subtypes.

**Suppl. Figure 2**

A) DNA and translated amino acid (aa) sequence of AHR KO organoid clones. B) Western blot image of nuclear extracts of three different control and AHR KO organoids clones. C) qPCR expression data of AHR target genes in AHR KO and control colon organoids treated with FICZ (4h). *, P < 0.05, **, P < 0.01, ***, P, < 0.001.

**Suppl. Figure 3**

A) Gene set enrichment plots of DEGs of the ENR CH-223191 vs. DMSO RNAseq relative to DEGs in UC patient organoids vs. controls^9^. B) Gene set enrichment plots of DEGs of the ENR CH-223191 vs. DMSO RNAseq relative to DEGs of either inflamed or non-inflamed epithelium of UC patient vs. healthy control (HC) samples^8^. C,D) qPCR expression data of overlapping genes from Fig.2G: C) d4 ENR UC and non-IBD adult patient organoids. D) 4 dENR control and AHR KO organoids.
